# Impact of active breaks on sedentary behavior and perception of productivity in office workers

**DOI:** 10.47626/1679-4435-2023-1213

**Published:** 2024-09-24

**Authors:** Leandra Batista-Ferreira, Daniel Dias Sandy, Paula Cristina Moreira C. Silva, Daniel José Matos Medeiros-Lima, Bernardo Minelli Rodrigues

**Affiliations:** 1 Faculty of Education and Health Sciences, University Center of Brasilia (CEUB), Brasilia, DF, Brazil; 2 Faculty of Medicine of Campos, Rio de Janeiro, RJ, Brazil; 3 Faculty of Medicine, Federal University of Rio de Janeiro, Rio de Janeiro, RJ, Brazil; 4 TAKE CARE BR - Lifestyle Medicine, Rio de Janeiro, RJ, Brazil; 5 Itaperuna University Foundation, Rio de Janeiro, Brazil

**Keywords:** sedentary behavior, occupational health, teleworking, sitting position, occupational medicine, comportamento sedentário, saúde ocupacional, teletrabalho, medicina do trabalho, postura sentada

## Abstract

**Introduction:**

The increasing prevalence ofsedentary behavior at work, which has been exacerbated by technological advancement and remote work models, can compromise worker health, leading to both physical and mental problems. Increasing research on sedentary behavior has resulted in interventions such as active breaks.

**Objectives:**

This study addresses the impact of sedentary behavior at work and the effects of active breaks.

**Methods:**

This descriptive-exploratory study with a mixed-methods approach included 70 professionals of both sexes, 86% women (35.2 [SD, 10.2] years) and 14% men (33.5 [SD, 11] years), who worked remotely in administrative roles. The intervention was a 25-week active break protocol involving lectures, a questionnaire, and an app.

**Results:**

At the end of the intervention, 64% of participants were taking active breaks. Spending > 10 hours a day in sedentary behavior reduced significantly (from 31% to 14%), as did the proportion of workers who did not exercise (from 43% to 26%; p = 0.002). There were also reductions in post-lunch sleepiness, perceived stress (p < 0.01), and pain/discomfort (p < 0.01).

**Conclusions:**

Management programs for sedentary behavior should consider the use of active breaks, since they can reduce sedentary behavior and perceived sleepiness, stress, and pain. This will result in a healthier work environment, increasing employee quality of life as well as company productivity.

## Abstract (pt)

**Introdução:**

Com a predominância do sedentarismo ocupacional, agravado pelo avanço tecnológico e pelo trabalho remoto, a saúde dos trabalhadores pode ser comprometida, incluindo problemas físicos e mentais, o que faz com que estudos sobre o comportamento sedentário e intervenções como pausas ativas ganhem destaque.

**Objetivos:**

O estudo aborda o impacto do sedentarismo no ambiente de trabalho e a relevância das pausas ativas para mitigar seus efeitos.

**Métodos:**

Tratou-se de estudo descritivo-exploratório com abordagem qualiquantitativa, realizado com 70 profissionais de ambos os sexos, 86% mulheres (35,2±10,2 anos) e 14% homens (33,5±11 anos). Todos trabalhavam remotamente em funções administrativas. Os participantes foram orientados a seguir uma rotina de pausas ativas durante 25 semanas. O estudo usou palestras, um questionário e um aplicativo para a prática.

**Resultados:**

Dos participantes, 64% adotaram as pausas ativas após a intervenção. Foi observada uma redução significativa no tempo sedentário (superior a 10 horas), de 31 para 14%, e no número de trabalhadores que não se exercitavam, de 43 para 26% (p = 0,002). Notou-se também uma redução na sonolência após o almoço, na percepção do estresse (p < 0,01) e nas dores e/ou desconforto no corpo (p < 0,01).

**Conclusões:**

A rotina de pausas ativas parece ser uma estratégia para diminuir o comportamento sedentário e melhorar a percepção quanto a sonolência, estresse e dores. Portanto, a implementação de programas de gestão ativa do comportamento sedentário, por meio de pausas ativas, pode proporcionar um ambiente de trabalho mais produtivo e saudável, beneficiando a qualidade de vida dos funcionários e a produtividade da empresa.

## INTRODUCTION

Sedentary behavior at work has been increasingly associated with both physical and mental health problems, largely due to prolonged mental demands and excessive sitting, which reduces metabolic activity and cellular oxygenation.^1,2^ The main causes of work absenteeism in Brazil are mental and behavioral disorders, osteoarticular disease, and chronic non-communicable disease.^3^ According to Rosenkranz et al.,^4^ their rate is higher among office workers, who spend a large part of their time sitting.

According to Souza et al.,^3^ Brazil is the most physically inactive country in Latin America and ranks fifth globally.^2,5^ The most recent data from the telephone disease surveillance system (VIGITEL Brasil) indicate that approximately 64% of Brazilians are sedentary or get insufficient exercise.^3^ Technological advancement in the twentieth century has led to a significant increase sedentary behavior and, thus, an increase in sedentarism-related diseases,^6^ including a high risk of cardiovascular and metabolic problems, in addition to early mortality.^7^ However, incorporating active breaks into the workday can mitigate these harmful health consequences.

### ACTIVE BREAKS

Active breaks are short rest periods in which workers perform movements designed to change their body posture and increase their heart rate.^1,7,8^ According to Thivel et al.,^6^ this procedure aims to prevent the emergence of disorders resulting from prolonged sitting, minimizing muscle and joint pain while improving blood circulation and activating the body as a whole.

Mama et al.^9^ found strong evidence that a lack of movement can negatively affect health, which underscores the need to consider intervention programs involving regular moderate-to-vigorous activity, including short active breaks during the workday, such as climbing stairs or any movement that increases energy expenditure and provides health benefits, in addition to more structured exercises during leisure time.

As society’s technological level has increased, physical effort has been reduced, a problem associated with the innate tendency to conserve energy and avoid unnecessary effort. The tendency to avoid energy expenditure may explain why people do not exercise regularly or move more frequently in the workplace, despite knowing the negative health effects of sedentarism.^6,10^ Thus, research on interventions for sedentary behavior has increased, leading to more realistic goals for stress management and overall well-being.^10^ “Move more, sit less” could be a clear and actionable message for intervention participants.^8^ In light of this, the present study investigated the effects of routine active work breaks on sedentary behavior and self-perceived vigor and occupational stress among office workers in an attempt to provide managers, leaders, and workers with relevant information for public and corporate policies to prevent sedentarism.

## METHODS

This qualitative and quantitative descriptive-exploratory study applied 2 rounds of structured questionnaires. The reference values for each item were adapted from reference and intervention articles. The questionnaires were administered remotely through an active break app.

A total of 70 office workers, 86% women (mean age 35.2 [SD, 10.2] years) and 14% men (mean age 33.5 [SD, 11] years), participated in the study. All participants were involved in a remote work model. The inclusion criteria were: providing written informed consent to participate (including data usage for the purposes of the study), age > 18 years, the ability to perform exercises independently, active employment in the participating company, completing the questionnaires, and registration on the app’s support platform (*Pausa Ativa Ocupacional*).

### PROCEDURES

The study was conducted over 25 weeks (December 2021 to June 2022) at a mental well-being and team development consultancy. The intervention began after an internal memo was circulated and a 60-minute awareness and guidance lecture was given remotely by a qualified specialist. All company employees were encouraged to register on the app platform and respond to the initial questionnaire (Table 1), as well as to download the app, which enabled them to participate in the intervention and receive support.

**Table 1:** Survey responses

Responses	Pre (n = 70)		Post (n = 70)		
	f	%	f	%	p-value
Sedentary behavior at work, hours per day					
> 10	22	31	10	14	0.001
8-10	24	34	26	37	
5-7	20	29	29	41	
1-4	4	6	5	7	
Weekly exercise frequency, times per week					
None	30	43	18	26	0.002
1	5	7	9	13	
2	13	19	7	10	
3	8	11	16	23	
4	5	7	2	3	
5	4	6	9	13	
6	3	4	5	7	
7	2	3	4	6	
Frequency of active breaks					
Never	45	64	3	4	< 0.001
Rarely	3	4	16	23	
Seldom (1 a day)	6	9	24	34	
Frequently (2-3 a day)	14	20	23	33	
Very frequently (≥ 4 a day)	2	3	4	6	
Post-lunch sleepiness					
Losing the fight against sleep, on the verge of falling sleep	0	0	0	0	0.002
Would like to lie down, but fighting sleep	0	0	4	6	
Drowsy, almost asleep, no interest in staying awake, slow thinking	4	6	5	6	
Sleepy but alert	16	24	4	6	
Relaxed, awake, responsive, but not completely alert	11	16	16	24	
Concentration not at maximum level	26	39	22	33	
Active, alert, and in a good mood	10	15	17	25	
Pain and discomfort					
Very often (≥ 4 times a week)	24	35	1	1	< 0.001
Frequently (2-3 times a week)	17	25	15	22	
Seldom (once a week)	27	39	30	43	
None	1	1	24	33	
Perceived stress					
Always	8	11	1	1	< 0.001
Frequently	11	16	5	7	
Occasionally	29	41	21	30	
Rarely	18	26	33	47	
Never	4	6	10	14	

f = frequency; n = number; Pre = before the intervention; Post = after the intervention.

**Figure 1:**
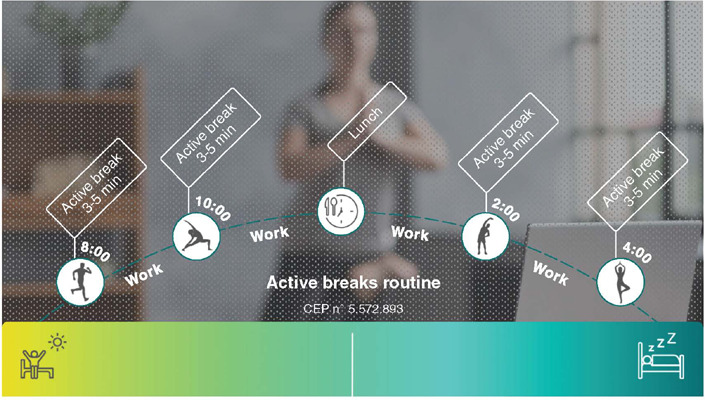
Active break intervention protocol.

After the launch and awareness lecture, a new wave of communication reinforced the study’s goals and encouraged registration on the app platform. The protocol consisted of taking active breaks at the following times: before beginning the work shift, mid-morning (preferably between 10:00 am and 10:30 am), after lunch (preferably between 2 pm and 2:30 pm), and late afternoon (preferably between 4 pm and 4:30 pm) (Figure 1). All volunteers were informed that the breaks were voluntary and were within their rights as workers (article 71 of the Consolidation of Labor Laws). They were encouraged to participate in sports or other daily exercise outside the work environment during their leisure time.

During the 24 weeks of intervention, an exclusive lecture was given to leaders and another was given to the entire team to reinforce the program. A forum for questions was provided via messaging application and telephone and was duly monitored by a physical education professional with experience in work-related physical activity and ergonomics. After the end of the 24th week, the company’s communications sector invited all employees to complete the questionnaire again to provide post-intervention data.

The study was approved by the institutional ethics committee (opinion no. 5,572,893) and was developed in accordance with Brazilian National Health Council Resolution 466/2012.^19^

### STATISTICAL ANALYSIS

The data were entered into a Microsoft Office Excel spreadsheet for subsequent analysis. The study population was characterized according to the investigated variables. Descriptive analysis and the Wilcoxon test were used to determine differences between pairs of the following variables: frequency of active breaks, sedentary behavior at work, and weekly exercise frequency. The McNemar test was used to compare the difference in proportions of post-lunch sleepiness, pain/discomfort; and perceived stress before and after the intervention. The significance level was set at 5%, p < 0.05. All statistical tests were performed in IBM SPSS Statistics 25.0. The results were analyzed and compared with data from the literature.

**Chart 1:** Participant questionnaire

	Score
1. Generally, how much time have you spent sitting, reclining, or lying down during your workday, Including commuting, breaks, and eating (breakfast, lunch, and supper)? (adapted from Tremblay et al.^11^ and Li et al.^12^)	
Hours a day	
> 10 8-10 5-7 1-4	( ) 1 ( ) 2 ( ) 3 ( ) 4
2. Do you regularly take 3- to 10-minute active breaks of during the workday (including walking, running, climbing stairs, lifting weights, stretching, dancing, or jumping)? (adapted from Hallgren et al.^13^ and Pedisic et al.^14^)	
Frequency	
Never Rarely Seldom (once a day) Frequently (2 to 3 times a day) Very frequently (4 or more times a day)	( ) 1 ( ) 2 ( ) 3 ( ) 4 ( ) 5
3. Over the last few weeks, how many times each week did you exercise or participate in sports for at least 20 consecutive minutes? (adapted from Li et al.^12^)	
Number of times	
0 1 2 3 4 5 6 7	( ) 1 ( ) 2 ( ) 3 ( ) 4 ( ) 5 ( ) 6 ( ) 7 ( ) 8
4. In recent weeks, how have you felt in the first few hours after lunch (post-lunch sleepiness)? (adapted from Hoddes et al.^15^)	
Condition	
Losing the fight against sleep, on the verge of falling sleep Would like to lie down, but fighting sleep Drowsy, almost asleep, no interest in staying awake, slow thinking Sleepy but alert Relaxed, awake, responsive, but not completely alert Concentration not at maximum level Active, alert, and in a good mood	( ) 1 ( ) 2 ( ) 3 ( ) 4 ( ) 5 ( ) 6 ( ) 7
5. How often, in the last few weeks, have you felt back pain or discomfort that could affect your work productivity? (adapted from Fairbank & Pynsent^16^ and Vigatto et al.^17^)	
Frequency	
Very often (≥ 4 times a week) Often (2-3 times a week) Rarely (once a week) Never	( ) 1 ( ) 2 ( ) 3 ( ) 4
6. Stress is a state feeling tense, restless, nervous, and anxious, affecting sleep due to one’s mind racing all the time. How frequently have you felt stressed in recent weeks? (adapted from Elo et al.^18^)	
Frequency	
Always Often Sometimes Rarely Never	( ) 1 ( ) 2 ( ) 3 ( ) 4 ( ) 5

## RESULTS

**Figure 2:**
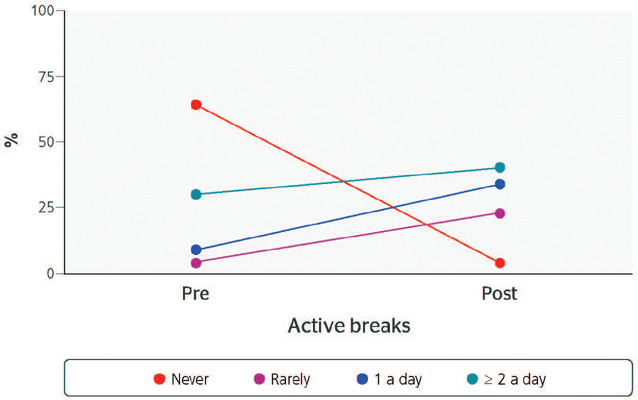
Frequency of active breaks before (Pre) and after (Post) the intervention.

The Wilcoxon test indicated significant increases in active breaks (p < 0.001) and weekly exercise (p = 0.002), as well as a significant reduction in sedentary behavior at work (p = 0.001). The proportion of workers who never took active breaks fell from 64% at baseline to 4% after the intervention. The proportion of those who rarely took active breaks rose from 4% at baseline to 23% after the intervention, and the proportion of those who took 1 active break each day increased from 9% at baseline to 34% after the intervention (Table 1) (Figure 2).

The proportion of workers who took at least 2 active breaks a day increased from 23% at baseline to 39% after the intervention (Table 1) (Figure 2), while the proportion of those who spent > 10 hours sitting during the work day reduced from 31% to 14%. Regarding weekly exercise, the proportion of those who did not participate in sports or get any type of exercise reduced from 43% at baseline to 26% after the intervention.

The McNemar test showed significant reductions in post-lunch sleepiness (p = 0.03), perceived stress (p < 0.01), and pain and/or discomfort (p < 0.01). Although the Wilcoxon test showed no significant difference between pairs of variables regarding post-lunch sleepiness (p = 0.2), the descriptive analysis showed reductions in post-lunch sleepiness (30% to 17%), reported pain/discomfort (60% to 23%) (p < 0.01), and perceived stress (68% to 39%) after the intervention (p < 0.01) (Table 1).

## DISCUSSION

We observed a significant change in sedentary behavior among these workers, which may have been related to introducing active breaks in their work routine. After 25 weeks, 60% of those who never took active breaks during their shift began taking them. This may be related to the findings of previous studies, ie, that active breaks benefit neurophysiological function, working memory, alertness, and perceived productivity.^20^ Kallings et al.^21^ and Pedisic et al.^14^ reported that active breaks restore the body and mind from the effects of periods of sitting. Thus, the significant change in behavior may stem from feeling these effects, since most had until that point never taken active breaks. This is in line with Spagnol,^22^ who highlighted the importance of implementing interventions during work shifts to promote worker productivity and well-being.

The positive effects of active breaks on the sample’s work routine can be seen in the 13% reduction in post-lunch sleepiness, the 37% reduction in pain/discomfort, and the 29% reduction in perceived stress. This could be related to positive physiological adaptations to short periods of intense physical activity, since, according to Heiland et al.,^23^ active movement that increase the heart rate for approximately 3 minutes can compensate for the negative effects of overload and low cerebral blood flow.

During active breaks, neural activation results in motor patterns that assimilate sensory input and coordinate the results of autonomic regulation, leading to anxiolytic and antidepressant effects, inhibiting excessive neural activity in the prefrontal cortex, and releasing hormones such as dopamine, serotonin and endorphins, which improve mood, reduce stress levels, and increase the ability to concentrate and focus.^12,24^ According to Tremblay et al.,^11^ sedentary behavior is characterized by any activity performed during waking hours that results in an energy expenditure ≤ 1.5 metabolic equivalents, whether in a sitting, reclining, or lying position.^11^ This is consistent with the results of previous research, which suggests that the amount of time spent sitting is an autonomous risk factor for health and disease development.

Studies show that spending > 5 hours a day in sedentary behavior harms health and reduces longevity. Sedentary periods > 8 hours a day are considered a significant risk factor for the emergence of noncommunicable diseases.^2,10,12^ However, according to Bull et al.,^2^ moderate-to-intense physical activity can maintain or even improve brain plasticity and result in changes in neuronal cells, reorganizing brain function and structures and improving tissue oxygenation. Thus, daily higher-intensity exercise, even for short periods, has been increasingly recommended.^2,24,25,26^ Our results regarding the reduction in post-lunch sleepiness corroborate these findings.

In 2021, the European Agency for Safety and Health at Work recommended that people should not spend > 50% of the work day sitting, should not sit for > 5 hours per day, should spend ≥ 10 minutes in movement for every 2 hours spent sitting, and should try to work actively by changing positions between sitting, standing, and walking. In 2015, the *British Journal of Sports Medicine* recommended that workers with sedentary/sitting roles should spend 2 hours a day standing and performing light activity (light walking) during work hours and take short, active breaks while standing.^10,22,26,27,28,29,30^ This research is relevant to our results, given that it explains the greater motivation and lower stress workers reported as they moved more both inside and outside the workplace.

## CONCLUSIONS

Our active break intervention in the workplace appears to be a viable strategy, positively affecting sedentarism and perceived sleepiness, stress, and pain among office workers. Such a routine can result in more active employees who are less reactive to work-related stress, thus promoting a healthier and more productive work environment.

We observed a trend away from sedentary behavior patterns, which significantly affected the workers’ behavior, perceived vitality, and physical, mental, and emotional resilience. These results indicate the relevance of including active breaks throughout the workday in sedentary behavior management programs, since productivity can be directly associated with sedentary behavior, in addition to the physical, mental, and emotional health of workers.

As study limitations, we point out the small sample size and the limited control of the break routine, since a remote work model was in place during the intervention, which restricts the generalizability of the results. Hence, future research on active break interventions should be conducted in more controlled remote work environments and involve greater in-depth analysis. This could contribute to new preventive interventions and health promotion strategies to improve worker quality of life and increase company productivity.
